# Supplementary material for: FERN – a Java framework for stochastic simulation and evaluation of reaction networks
Source: BMC Bioinformatics. 2008 Aug 29;9:356. doi: 10.1186/1471-2105-9-356 (PMC2553347; doi:10.1186/1471-2105-9-356)
Supplement: Additional file 1 — FERN distribution, Version 1.3. This archive contains the FERN source code and binaries as well as documentation and example models in FernML and SBML. [file 1471-2105-9-356-S1.zip › fern/doc/javadoc/fern/tools/gnuplot/TransposedArrayMatrixAxes.html]

TransposedArrayMatrixAxes


---


|  |  |  |
| --- | --- | --- |
| **Overview** \| **Package** \| **Class** \| **Use** \| **Tree** \| **Deprecated** \| **Index** \| **Help** | |  |
| **PREV CLASS**   NEXT CLASS | **FRAMES**    **NO FRAMES**     **All Classes** |  |
| SUMMARY: NESTED \| FIELD \| CONSTR \| METHOD | DETAIL: FIELD \| CONSTR \| METHOD |  |


---


## fern.tools.gnuplot Class TransposedArrayMatrixAxes

```
java.lang.Object
  fern.tools.gnuplot.Axes
      fern.tools.gnuplot.ArrayMatrixAxes
          fern.tools.gnuplot.TransposedArrayMatrixAxes
```

**All Implemented Interfaces:**: Iterable<String>

---

``` public class TransposedArrayMatrixAxes extends ArrayMatrixAxes ```

Implementation of `Axes` to use with transposed array matrixes
of any number type (the type of the arrays is inferred by
using the reflection api). An array matrix is just a two dimensional
rectangular array (`new double[20][3]`). The first index
of the transposed matrix gives the column, the second the row (3 rows and 20 columns).

If your array matrix is not transposed (your first index denotes the row, the second
one the column), just use `ArrayMatrixAxes`.

**Author:**
:   Florian Erhard

**See Also:**: `Axes`

---

**Field Summary**

**Fields inherited from class fern.tools.gnuplot.ArrayMatrixAxes**

`array`


**Constructor Summary**

`TransposedArrayMatrixAxes(Object matrix)`
  
          Create a new `Axes` object containing the given array matrix
without labels / styles.

`TransposedArrayMatrixAxes(Object matrix,
String[] labels,
String[] styles)`
  
          Create a new `Axes` object containing the given array matrix
with the given labels /styles (either can be `null`).


| **Method Summary** | |
| --- | --- |
| `int` | `getNumColumns()`             Gets the number of columns. |
| `int` | `getNumRows()`             Gets the number of rows. |
| `Iterator<String>` | `iterator()`             Yields the tab separated columns row by row. |

**Methods inherited from class fern.tools.gnuplot.ArrayMatrixAxes**

`checkMatrix`

**Methods inherited from class fern.tools.gnuplot.Axes**

`addAxes, applyDefaultStyle, getAdditionalAxesIterator, getLabel, getNumAdditionalColumns, getStyle, loadLabelsAndLineStyles, setLabel, setStyle`

**Methods inherited from class java.lang.Object**

`clone, equals, finalize, getClass, hashCode, notify, notifyAll, toString, wait, wait, wait`

**Constructor Detail**

### TransposedArrayMatrixAxes

```
public TransposedArrayMatrixAxes(Object matrix)
```

:   Create a new `Axes` object containing the given array matrix
    without labels / styles. If the passed object is not a valid array matrix,
    an `IllegalArgumentException` will be thrown.

    **Parameters:**: `matrix` - array matrix

---


### TransposedArrayMatrixAxes

```
public TransposedArrayMatrixAxes(Object matrix,
                                 String[] labels,
                                 String[] styles)
```

:   Create a new `Axes` object containing the given array matrix
    with the given labels /styles (either can be `null`).
    If the passed object is not a valid array matrix,
    an `IllegalArgumentException` will be thrown.

    **Parameters:**: `matrix` - the array matrix: `labels` - the labels: `styles` - the styles


**Method Detail**

### getNumRows

```
public int getNumRows()
```

:   **Description copied from class: `Axes`**
:   Gets the number of rows.

    :   **Overrides:**: `getNumRows` in class `ArrayMatrixAxes`
    :   **Returns:**: number of rows.

---


### getNumColumns

```
public int getNumColumns()
```

:   **Description copied from class: `Axes`**
:   Gets the number of columns. The number of the attached `Axes`
    object is also included.

    :   **Overrides:**: `getNumColumns` in class `ArrayMatrixAxes`
    :   **Returns:**: number of columns.

---


### iterator

```
public Iterator<String> iterator()
```

:   Yields the tab separated columns row by row.

    :   **Specified by:**: `iterator` in interface `Iterable<String>` **Overrides:**: `iterator` in class `ArrayMatrixAxes`


---


|  |  |  |
| --- | --- | --- |
| **Overview** \| **Package** \| **Class** \| **Use** \| **Tree** \| **Deprecated** \| **Index** \| **Help** | |  |
| **PREV CLASS**   NEXT CLASS | **FRAMES**    **NO FRAMES**     **All Classes** |  |
| SUMMARY: NESTED \| FIELD \| CONSTR \| METHOD | DETAIL: FIELD \| CONSTR \| METHOD |  |


---
